# Comparative observation between Roxithromycin and Azithromycin sequential therapy in the treatment of Mycoplasma pneumoniae Pneumonia in Children

**DOI:** 10.12669/pjms.40.9.8942

**Published:** 2024-10

**Authors:** Dan Liu, Yi-fei Zhang

**Affiliations:** 1 Dan Liu Department of Neonatology, Associate Chief Physician, Associate Professor, The First Affiliated Hospital of Yangtze University, Jingzhou 434000, Hubei, China. The First Clinical Medical Collage of Yangtze University, Jingzhou 434000, Hubei, China; 2 Yi-fei Zhang Department of Pediatric Medicine, Chief Physician, The First Affiliated Hospital of Yangtze University, Jingzhou 434000, Hubei, China

**Keywords:** Children, Mycoplasma pneumoniae pneumonia, Roxithromycin, Azithromycin, Sequential therapy, Clinical efficacy

## Abstract

**Objective:**

To investigate the clinical efficacy of roxithromycin combined with azithromycin sequential therapy in the treatment of mycoplasma pneumoniae pneumonia in children.

**Methods:**

A retrospective study was conducted on 100 patients with mycoplasma pneumoniae pneumonia admitted to The First Affiliated Hospital of Yangtze University from January 2020 to December 2022. All patients were divided into the observation group (roxithromycin combined with azithromycin sequential therapy) and the control group (azithromycin sequential therapy), with 50 cases in each group. The clinical efficacy, improvement time of clinical symptoms/signs, inflammation indexes, oxidative stress indexes and immune function levels of the two groups were compared. Moreover, the improvement of lung function indexes and the adverse reactions were observed.

**Results:**

The overall response rate of the observation group was 96.00%, which was higher than the control group (84.00%) (*p<*0.05). The time of clinical symptoms/signs in the observation group were significantly lower than those in the control group(*p<*0.05). After treatment, significant improvements were seen in the levels of CRP, TNF-ɑ, IL-6, GSH-Px, SOD, MDA, CD3^+^, CD4^+^, CD4^+^/CD8^+^, IgM, IgG , IgA, FEV_1_, FEV_1_%, FVC and FEV_1_/FVC of the two groups compared with those before treatment (*p<*0.05), and the improvement in the observation group was more obvious than that in the control group(*p<*0.05). The overall incidence of adverse reactions in the observation group was 6.00%, which was slightly lower than that in the control group (8.00%) (c²=0.154, *P*=0.695).

**Conclusion:**

Roxithromycin combined with azithromycin sequential therapy is a safe regimen for the treatment of mycoplasma pneumoniae pneumonia in children.

## INTRODUCTION

Mycoplasma pneumoniae pneumonia, as a common atypical community-acquired pneumonia in children’s respiratory tract, is mostly seen in children under 12 years old.^1^,^2^ Its clinical manifestations are diversified, if the course of disease exceeds four weeks, it will develop into persistent mycoplasma pneumoniae pneumonia. Failure to receive timely and effective treatment may lead to serious consequences, such as respiratory distress and death, causing serious harm to the life and health of children.^3^,^4^ When treating the disease, drug therapy is preferred and anti-infective therapy is taken as the principle.^5^ Macrolide antibiotics are the primary drugs to treat mycoplasma pneumoniae pneumonia. Azithromycin sequential therapy, as a conventional method for the treatment of mycoplasma pneumoniae pneumonia in children, shows satisfactory clinical efficacy.

However, azithromycin alone has limited efficacy. If the dosage is increased, adverse reactions will increase correspondingly. Combined use is a trend in the clinical treatment of mycoplasma pneumoniae pneumonia at present. Considering their close correlation with the occurrence and development of mycoplasma pneumoniae pneumonia, the improvement of oxidative stress and immune function is helpful in the treatment of mycoplasma pneumoniae pneumonia. Therefore, in this study to observe the efficacy and safety of roxithromycin combined with azithromycin sequential therapy in the treatment of mycoplasma pneumoniae pneumonia in children.

## METHODS

This was a retrospective study. Children with mycoplasma pneumoniae pneumonia admitted to The First Affiliated Hospital of Yangtze University from January 2020 to December 2022 were selected as subjects and divided into two groups according to treatment methods: the observation group and the control group, with 50 cases in each group.

### Ethical Approval:

The study was approved by the Institutional Ethics Committee of The First Affiliated Hospital of Yangtze University (No.: LL2023126; date: July 22,2023), and written informed consent was obtained from all participants’ guardians.

### Inclusion criteria:

- Children who met the relevant diagnostic criteria of mycoplasma pneumoniae pneumonia in the Expert Consensus on Diagnosis and Treatment of Mycoplasma pneumoniae pneumonia in Children (2015 Edition).
- Those diagnosed by etiology (The particle agglutination method was used to detect antibodies against mycoplasma pneumoniae, and the result was higher than 1:160) and X-ray examination; those with cough, fever, chest pain and other symptoms.
- Who had not received relevant treatment before enrollment.
- Those whose guardians agreed to participate in the study and signed an informed consent form.

### Exclusion criteria:

- Children with contra indications or allergic to the drugs in this study.
- Those with immune deficiency or serious infectious diseases.
- Those with hepatic and renal insufficiency or systemic diseases.
- Those with other lung or respiratory diseases, such as tuberculosis or asthma.
- Those with poor compliance and difficulty in completing the study.

Design a clinical sample size estimation formula based on differential studies, N=[p1 x (1-p1)+p2 x (1-p2) x (μa/2+μb) ^2^]/(p1-p2) ^2^, take α=0.05, β=0.1,In the early stage of this study, the observation group had an effective rate of >95%, while the control group had an effective rate of >70%, The calculation formula with p1=0.95 and p2=0.70 yields a result of 45. Considering a 10% dropout rate, at least 49 cases should be collected from each group.

All children were given conventional symptomatic treatment. The control group were treated with azithromycin sequential therapy: azithromycin for injection was added into 250 ml of 0.9% sodium chloride injection by intravenous drip once a day, and it was treated continuously for 3-7 days according to the severity of the disease. After three days of withdrawal, it was changed to oral azithromycin dry suspension of 10 mg/(kg·d) once a day, and stopped for four days after three consecutive days of treatment, a total of two weeks. The observation group were given roxithromycin combined with azithromycin sequential therapy, with roxithromycin 10 mg/(kg·d) taken orally twice, take it continuously for 6 days, and switch to azithromycin after the patient’s condition stabilizes, the azithromycin was taken in the same way as the control group.

### Observation indicators:

- Clinical efficacy and improvement time of clinical symptoms/signs: The evaluation criteria for efficacy: Cured: complete disappearance of clinical symptoms/signs, and disappearance of the lesion on chest X-ray examination; Markedly effective: basic disappearance of clinical symptoms/signs, and majority absorption of the lesion on chest X-ray examination; Effective: slight improvement in clinical signs/symptoms, and partial absorption of lesions on chest X-ray examination. Invalid: no improvement in clinical signs/symptoms, and no absorption of the lesion on chest X-ray examination. Overall response rate= (cured+markedly effective + effective)/total cases × 100%. The improvement time of symptoms/signs included body temperature recovery time, asthma relief time, cough relief time, lung rales disappearance time and length of stay.
- Inflammatory indexes, oxidative stress indexes and immune function levels. Venous blood was drawn before treatment and three days after all treatment in the fasting state. The levels of serum C-reactive protein (CRP), tumor necrosis factor-α(TNF-α) and Interleukin-6 (IL-6) were detected by double-antibody sandwich ELISA. Glutathione peroxidase (GSH-Px), superoxide dismutase (SOD) and malondialdehyde (MDA) were determined by chemical colorimetry with the automatic biochemical analyzer. T lymphocyte subsets in peripheral blood, including CD3^+^, CD4^+^ and CD4^+^/CD8^+^ levels, were detected by flow cytometry. Moreover, immunoglobulin M (IgM), immunoglobulin G (IgG) and immunoglobulin A (IgA) were detected by colloidal gold method.
- The lung function indexes: forced expiratory volume in one second (FEV_1_), the percentage of forced expiratory volume in the predicted value in the first second (FEV_1_%), forced vital capacity (FVC) and FEV_1_/FVC level.
- The adverse reactions during the treatment were recorded and compared.

### Statistical Analysis:

All data in this study were statistically analyzed using SPSS22.0 software, and measurement data were expressed as (*χ̅*±*S*). The confidence interval was 95%. T-test was utilized for inter-group comparison, and paired T-test was used for intra-group mean comparison before and after treatment. χ^2^ test was used to compare rates, and *p<*0.05 was considered to have a statistically significant difference.

## RESULTS

In the control group, there were 28 males and 22 females, aged from 6 to 12 years, with an average of (8.62±1.88) years. The course of the disease was (3.08±1.58) weeks; Lesion location: 21 cases were on the left side, 18 cases on the right side and 11 cases on both sides; In addition, there were 38 mild cases and 12 severe cases. In the observation group, there were 30 males and 20 females, aged from 6 to 12 years, with an average of (8.52±2.00) years. The course of the disease was (3.04±1.63) weeks; Lesion location: 22 cases were on the left side, 16 cases on the right side and 12 cases on both sides; In addition, there were 35 mild cases and 15 severe cases. No statistically significant difference was observed in the comparison of general data between the two groups (*p >*0.05), which was comparable.

The overall response rate of the observation group was 96.00%, which was higher than the control group (84.00%), with a statistically significant difference (*p<*0.05), Table-I. The symptoms/signs in the observation group were significantly lower than those in the control group, with statistically significant differences (*P* <0.05), Table-II.

**Table-I:** Comparison of clinical efficacy between two groups [n (%)].

Group	n	Cured	Markedly effective	Effective	Invalid	Overall response rate (%)
Observation group	50	34 (68.00)	10 (20.00)	4 (8.00)	2 (4.00)	48 (96.00)
Control group	50	25 (50.00)	15 (30.00)	2 (4.00)	8 (16.00)	42 (84.00)
χ^2^ value						4.000
P value						0.046

**Table-II:** Comparison of the improvement time of symptoms/signs between two groups (*χ̅*±*S*).

Group	n	Body temperature recovery time(d)	Asthma relief time(d)	Cough relief time(d)	Lung rales disappearance time(d)	Length of stay(d)
Observation group	50	3.82±0.98	2.30±0.51	3.76±0.80	6.18±1.52	7.12±0.96
Control group	50	4.26±0.56	2.62±0.75	4.46±0.99	7.18±1.38	10.58±1.11
t value		2.744	2.496	3.885	3.442	16.677
P value		0.007	0.014	0.000	0.001	0.000

After treatment, significant improvements were seen in the levels of CRP, TNF-ɑ, IL-6, GSH-Px, SOD, MDA, CD3^+^, CD4^+^, CD4^+^/CD8^+^, IgM, IgG and IgA of the two groups compared with those before treatment (*p<*0.05), and the improvement in the observation group was more obvious than that in the control group(*p<*0.05), Table-III. After treatment, significant improvements were seen in the levels of FEV_1_, FEV_1_%, FVC and FEV_1_/FVC in the two groups compared with before treatment, and the improvement in the observation group was better than that in the control group(*p<*0.05), Table-IV.

**Table-III:** Comparison of inflammatory factors, oxidative stress and immune function between the two groups before and after treatment (*χ̅*±*S*)

Observation indicators	Observation point	Observation group	Control group	t	p
CRP (mg/L)	Before treatment	37.59±3.51	36.96±2.43	1.045	0.299
	After treatment	13.76±2.39	18.92±3.09	9.337	0.000
TNF-ɑ (ng/L)	Before treatment	1.75±0.18	1.74±0.17	0.465	0.643
	After treatment	1.10±0.10	1.28±0.06	10.610	0.038
IL-6 (ng/L)	Before treatment	112.18±7.63	111.79±5.86	0.290	0.773
	After treatment	53.16±5.58	57.38±5.25	3.898	0.000
GSH-Px (U/L)	Before treatment	18.52±2.32	18.59±3.09	0.125	0.901
	After treatment	37.40±3.51	35.73±3.41	2.411	0.018
SOD (NU/L)	Before treatment	60.60±4.15	60.62±4.19	0.024	0.981
	After treatment	66.30±3.84	63.22±3.72	4.073	0.000
MDA (nmol/mL)	Before treatment	10.66±3.83	10.75±3.23	0.121	0.904
	After treatment	5.61±2.49	6.74±2.64	2.198	0.030
CD3^+^(%)	Before treatment	41.80±6.94	43.11±6.44	0.974	0.333
	After treatment	47.72±6.89	43.52±6.40	3.160	0.002
CD4^+^(%)	Before treatment	27.66±4.31	28.08±3.79	0.508	0.613
	After treatment	38.61±5.09	34.60±4.61	4.135	0.000
CD4^+^/CD8^+^(%)	Before treatment	1.28±0.10	1.27±0.07	0.533	0.595
	After treatment	1.75±0.19	1.54±0.15	6.091	0.000
IgM (g/L)	Before treatment	1.41±0.66	1.38±0.73	0.229	0.819
	After treatment	2.77±0.60	2.37±0.47	3.701	0.000
IgG (g/L)	Before treatment	1.37±0.27	1.30±0.30	1.085	0.281
	After treatment	2.85±0.77	2.36±0.48	3.851	0.000
IgMA (g/L)	Before treatment	1.36±0.28	1.30±0.29	1.010	0.315
	After treatment	2.82±0.77	2.27±0.42	4.449	0.000

**Table-IV:** Comparison of lung function (*χ̅*±*S*).

Observation indicators	Observation point	Observation group	Control group	t	p
FEV_1_ (L)	Before treatment	1.38±0.29	1.30±0.30	1.306	0.195
	After treatment	2.21±0.26	1.68±0.24	10.560	0.000
FEV_1_ %	Before treatment	41.84±4.33	42.26±3.99	0.497	0.620
	After treatment	64.73±5.44	56.75±4.94	7.678	0.000
FVC (L)	Before treatment	2.32±0.66	2.25±0.71	0.484	0.630
	After treatment	2.87±0.68	2.50±0.47	3.099	0.003
FEV_1_/FVC	Before treatment	0.64±0.22	0.64±0.24	0.067	0.946
	After treatment	0.80±0.18	0.68±0.12	3.867	0.000

During the treatment, including two cases of nausea and one case of dizziness in the observation group, with a total incidence of adverse reactions of 6.00%. There were two cases of nausea and two cases of dizziness in the control group, with a total incidence of adverse reactions of 8.00%. No statistically significant difference was observed in the total incidence of adverse reactions between the two groups (c² =0.154, *P*=0.695).

## DISCUSSION

This study used sequential treatment with erythromycin combined with azithromycin to treat Mycoplasma pneumonia in children. The results showed that the total effective rate of the observation group was 96.00%, which was higher than the control group’s 84.00% (*p<*0.05). The time for temperature recovery, asthma relief, cough relief, lung rale disappearance, and hospital stay in the observation group were significantly lower than those in the control group (*p<*0.05), but there was no significant difference in the total incidence of adverse drug reactions between the two groups(o²=0.154), *p*=0.695). Research suggests that the sequential treatment of erythromycin combined with azithromycin in children with mycoplasma pneumonia has good clinical efficacy and high safety. This is consistent with the results of multiple studies.^6^,^7^ The reason why sequential treatment of both drugs has better clinical efficacy than single drug therapy is that their mechanisms of action are the same.

Mycoplasma pneumoniae pneumonia is an acute lower respiratory tract infectious diseases caused by mycoplasma pneumoniae infection,^6^ which has a high incidence in pediatrics and seriously threatens the life and physical and mental health of children.^8^ Research has shown that,^9^,^10^ mycoplasma pneumoniae damages the respiratory mucosa, triggers inflammatory reactions, releases inflammatory mediators, promotes the production of allergic reactions, and enhances immune response. Mycoplasma is highly sensitive to antibiotics and is often treated with macrocyclic phenolic antibiotics in clinical practice.^11^ Currently, azithromycin and roxithromycin are commonly used macrolide antibiotics in clinical practice.^12^ Sequential therapy is a new type of antibiotic medication model that can reduce treatment cycles, improve clinical efficacy, and reduce complications such as pain and infection caused by intravenous injection.^13^,^14^

Roxithromycin has a first dose effect, and its distribution characteristics in the body conform to the two compartments model. The effect of action is time-dependent, and the time for clinical symptom improvement is slow.^15^ On the other hand, azithromycin has a higher concentration in lung tissue, strong treatment targeting, and a longer half-life. The advantages of both drugs complement each other, thereby improving clinical treatment effectiveness.^16^ However, long-term use of azithromycin can lead to drug resistance and even affect the physical development of children, so sequential treatment can also reduce and avoid the above-mentioned side effects.

As shown in this study, the levels of inflammatory factors, oxidative stress indexes and immunoglobulin in the serum of children in the observation group were significantly improved after treatment, among which the levels of CD3^+^, CD4^+^, CD4^+^/CD8^+^ and IgM, IgG and IgA were significantly higher than those in the control group (*p<*.05). The levels of CRP, TNF-ɑ and IL-6 were significantly lower than those before treatment, and the level of reduction in the observation group was more obvious than that in the control group (*p<*0.05). Besides, the levels of GSH-Px, SOD and MDA in the observation group were better than those in the control group (*p<*0.05). This is similar to the results of multiple studies.^17^,^18^ The reasons may be as follows: mycoplasma pneumoniae pneumonia will have immune disorder, and the release of inflammatory factors will lead to a systemic inflammatory reaction. In addition, the occurrence and development of mycoplasma pneumoniae pneumonia are also obviously related to oxidative stress.^19^,^20^

As shown in this study, significant improvements were seen in the levels of FEV_1_, FEV_1_%, FVC and FEV_1_/FVC in the two groups compared with before treatment, and the improvement in the observation group was better than the control group (*p<*0.05). This fully demonstrated that roxithromycin combined with azithromycin sequential therapy was beneficial to the recovery of lung function in children.^21^

### Limitations:

It includes the small number of observed cases and the short follow-up period. In future more patients need to be included in follow-up studies, and follow-up time should be extended to further evaluate the efficacy of the treatment regimen.

## CONCLUSIONS

To sum up, roxithromycin combined with azithromycin treatment of mycoplasma pneumoniae pneumonia in children, can improving the immune function of children, reducing the oxidative stress response and inflammatory response, and ameliorating the lung function. It has satisfactory efficacy while less adverse drug reactions.

### Authors’ Contributions:

**YZ:** Carried out the studies, data collection, drafted the manuscript, are responsible and accountable for the accuracy or integrity of the work.

**DL:** Performed the statistical analysis and participated in its design.

**YZ** and **DL:** Participated in acquisition, analysis, or interpretation of data and draft the manuscript. All authors read and approved the final manuscript.
